# Characterization of the apical bridge barrier formed following amelogenin apexification

**DOI:** 10.1186/s12903-018-0641-0

**Published:** 2018-12-04

**Authors:** Maha M. F. Mounir, Jamila M. A. Farsi, Turki Y. Alhazzazi, Moustafa A. Matar, Azza A. El-Housseiny

**Affiliations:** 1 Department of Diagnostic Oral Sciences, King Abdulaziz University, Faculty of Dentistry, Jeddah, Saudi Arabia; 2 Department of Oral Biology, Faculty of Dentistry, Alexandria University, Alexandria, Egypt; 3 Department of Oral Biology, King Abdulaziz University, Faculty of Dentistry, Jeddah, Saudi Arabia; 4 Department of Pediatric Dentistry, Faculty of Dentistry, Pharos University, Alexandria, Egypt; 5 Pediatric Dentistry Department, Faculty of Dentistry, King Abdulaziz University, Jeddah, Saudi Arabia; 6 Pediatric Dentistry Department, Faculty of Dentistry, Alexandria University, Alexandria, Egypt

**Keywords:** Recombinant amelogenin, RAP, R-amelogenin, Regeneration, Apexification, Root canal therapy, Non-vital teeth, Open apex, Immature teeth

## Abstract

**Background:**

Recombinant amelogenin protein (RAP) is reported to induce complete root apex formation in dog model when used as apexification therapy. It also induces pulp regeneration in 85% of the treated group. Thus, the aim of this study was to investigate the nature of the remaining regenerated calcified tissues of the RAP group that showed no pulp regeneration compared to the calcium hydroxide treated group (CH).

**Methods:**

A total of 240 dogs’ open apex root canals were used, after establishment of canals contamination. Canals were cleaned, irrigated, and filled with RAP as an apexification material and compared with CH. Treated teeth were assessed by H&E, trichrome staining, and/or immunohistochemistry technique, at 1, 3, and 6 months.

**Results:**

A time-dependent increase in the calcified tissue barrier was observed in the apex of the RAP-treated group compared to the CH-treated group. The newly formed dentin in this RAP group was mainly tubular dentin and was functionally attached to the bone by periodontal ligament, while the CH group showed dentin-associated mineralized tissue (DAMT) associated with the newly formed apical barrier.

**Conclusions:**

Out results suggest that RAP can be used as novel apexification material, resulting in a thickening and strengthening of the canal walls, and achieving apical closure.

## Background

When teeth with incomplete root formation suffer pulpal necrosis, root development ceases, the canal remains large with thin or fragile walls, and the apex remains open [1]. These features make canals instrumentation and the formation of an adequate apical stop difficult. This requires the placement of an intracanal medicament to stimulate apical healing and formatting an apical barrier (AB) [2]. The commonly accepted medicament for apexification is calcium hydroxide (CH). Although depending on the clinical application, some demonstrated that CH show the least biocompatibility effect when used as a root canal filling material in dog deciduous teeth compared to Maisto (paste) and sealer 26 with iodoform [3], others show that CH can successfully induce apical closure in young permanent incisors with necrotic teeth and resolve clinical symptoms [4]. However, CH therapy has many disadvantages, including variability of treatment time, unpredictability of apical closure, and patient compliance [5]. In addition, long-term CH therapy has been shown to make teeth brittle [6]. In contrast, mineral trioxide aggregate (MTA) has also been used to provide an artificial barrier. It has the advantage of more predictable outcomes, less treatment time, and relies less on patient compliance. However, MTA still has limitations, including its inability to reinforce the root canal dentin and its high cost [7]. This implicates the need of a new regenerative material that can overcome these biological and technical disadvantages.

Amelogenin is an extracellular matrix protein that regulates the initiation and growth of hydroxyapatite crystals during mineralization of enamel [8] and also directs the formation of cementum during embryonic root development [9, 10]. Amelogenin splicing isoforms, leucine-rich amelogenin peptide (LRAP), induces osteogenesis in various cell types by activating the canonical Wnt signaling pathway to induce osteogenic differentiation [11]. LRAP treatment induces significant increases in mineral matrix formation and in bone sialoprotein and osterix gene expression. In addition, the impaired osteogenesis of amelogenin-null ES cells is partially rescued by the addition of exogenous LRAP [12, 13].

Recently, the capacity of the recombinant amelogenin protein (rM180) to act as an apexification therapy, to facilitate incomplete root apex formation in a dog model [14]. Amelogenin-treated canals showed calcified tissue formation at the apical foramen that was functionally attached to bone by an oriented periodontal ligament in 89.2% of the specimens [14]. Additionally, this treatment also induced pulp regeneration in 85% of the treated canals. Canals that showed no pulp regeneration still showed thickened root walls covered by an odontoblastic cell layer in addition to the closed apex.

This study investigated the nature of the regenerated calcified tissues of the RAP group that showed no pulp regeneration compared to the no pulp regeneration group treated with calcium hydroxide (CH).

## Methods

### Animals preparation

All procedures details were previously mentioned and described by Mounir et al. [14]. In brief, a total of 24 mongrel dogs of 6 months of age were included in this study. Animals were maintained and observed for health assessment before any endodontic procedures were performed.

### Canal preparation

After anesthetizing animals and pre-operative radiographs confirmed the presence of open apices in the mandibular and maxillary premolars, endodontic access was performed, and pulp tissue was completely removed using H-files. Teeth were managed and left without coronal restoration for 14 days to allow contamination [14, 15]. Animals were then anesthetized using sodium pentobarbital intravenous injection (30 mg/kg body weight); canals were cleaned under aseptic conditions to within 1 mm of the radiographic apices using large H-files in gentile filing movement and 1% sodium hypochlorite was used as an irrigant. After drying the canals with sterile paper points, a cotton pellet was placed in the pulp chamber then sealed with a temporary filling for 7 days (Orafil G, Colostol, Fermin, India). Teeth that showed no signs of infection were included in the study. A total of 240 root canals were divided into two groups: a recombinant amelogenin protein (180 amino acid mouse amelogenin) (RAP) group and the calcium hydroxide-treated (CH) group. An intermediate restorative material (IRM) was then carefully placed over the root canal dressing, and then access cavity was restored with amalgam. After 1, 3, and 6 months postoperatively, the animals were euthanized by intravenous (IV) injection of 20% Pentobarbitone solution, then the samples were evaluated.

### Histological procedures and immunohistochemistry protocol

Prepared specimens were stained using hematoxylin and eosin (H&E), trichrome stain, and different immunohistochemical stains. Antibodies for Wnt 10b and nestin antibodies were obtained from Abcam (Cambridge, UK, ab91201 and ab105389, respectively). All steps were performed following the manufacturers’ instructions. Conjugated secondary antibodies were obtained from Thermo Fisher Scientific (Fremont, CA USA). Sections were imaged using a Nikon Eclipse 80i microscope (Tokyo, Japan).

### Statistical analysis

This study was based on an initial sample size of 240 canals harvested and observed over 3 time intervals of 1, 3 and 6 months. There were some canals excluded from the study by selection criteria and others that were lost during processing. These values are shown in Table 1. The summative outcome of this study are described in Tables 1 and 2. Chi-Square test was used to compare the two proportions with significant differences set at *p* < 0.05 level.

**Table 1:** Sample Distribution Overview

	Number	%
Canals	240	100.0
Time Distribution		
1 month	80	33.3
3 months	80	33.3
6 months	80	33.3
Group Distribution		
Amelogenin	120	50.0
Calcium hydroxide (Ca (OH)2)	120	50.0
Root Closure		
Complete	98	40.8
Incomplete	71	29.6
None	49	20.4
Missing	22	9.2
Presence of DAMT		
Amelogenin	0.0	0.0
(Ca (OH) 2)	52	43.3
Presence of Calcified islands CIs		
Amelogenin	6.0	5.0
(Ca (OH) 2)	28	23.3

(DAMT) dentin-associated mineralized tissue

**Table 2:** Statistical Analysis of Treatments outcome after six months

Canal Variable	Number (%)	Treatment		*p*-value
		Amelogenin	Ca (OH) 2	
Canals	240 (100.0)	120 (50.0)	120 (50.0)	–
Root Closure				
Complete	98 (40.8)	85 (70.9)	13 (10.8)	< 0.001*
Incomplete	71 (29.6)	22 (18.3)	49 (40.8)	
None	49 (20.4)	10 (8.3)	39 (32.5)	
Presence of DAMT				
Yes	52 (21.6)	0.0 (0.0)	52 (43.3)	< 0.001*
Presence of CIs				
Yes	34 (14.1)	6 (5.0)	28 (23.3)	< 0.001*

*Significant using Chi-Square test *P* < 0.05 level

(CIs) Calcified islands

(DAMT) dentin-associated mineralized tissue

## Results

In a previous work, we showed that nearly 85% of the RAP-treated group showed pulp regeneration, the rest did not show pulp regeneration and none of the CH-treated group showed pulp regeneration as well [14]. Thus, a comparison of the nature of the remaining regenerated calcified tissues of the RAP-treated group that showed no pulp regeneration compared to the CH-treated group would be described below.

### Calcium hydroxide-treated-canal cohort

**Fig. 1:**
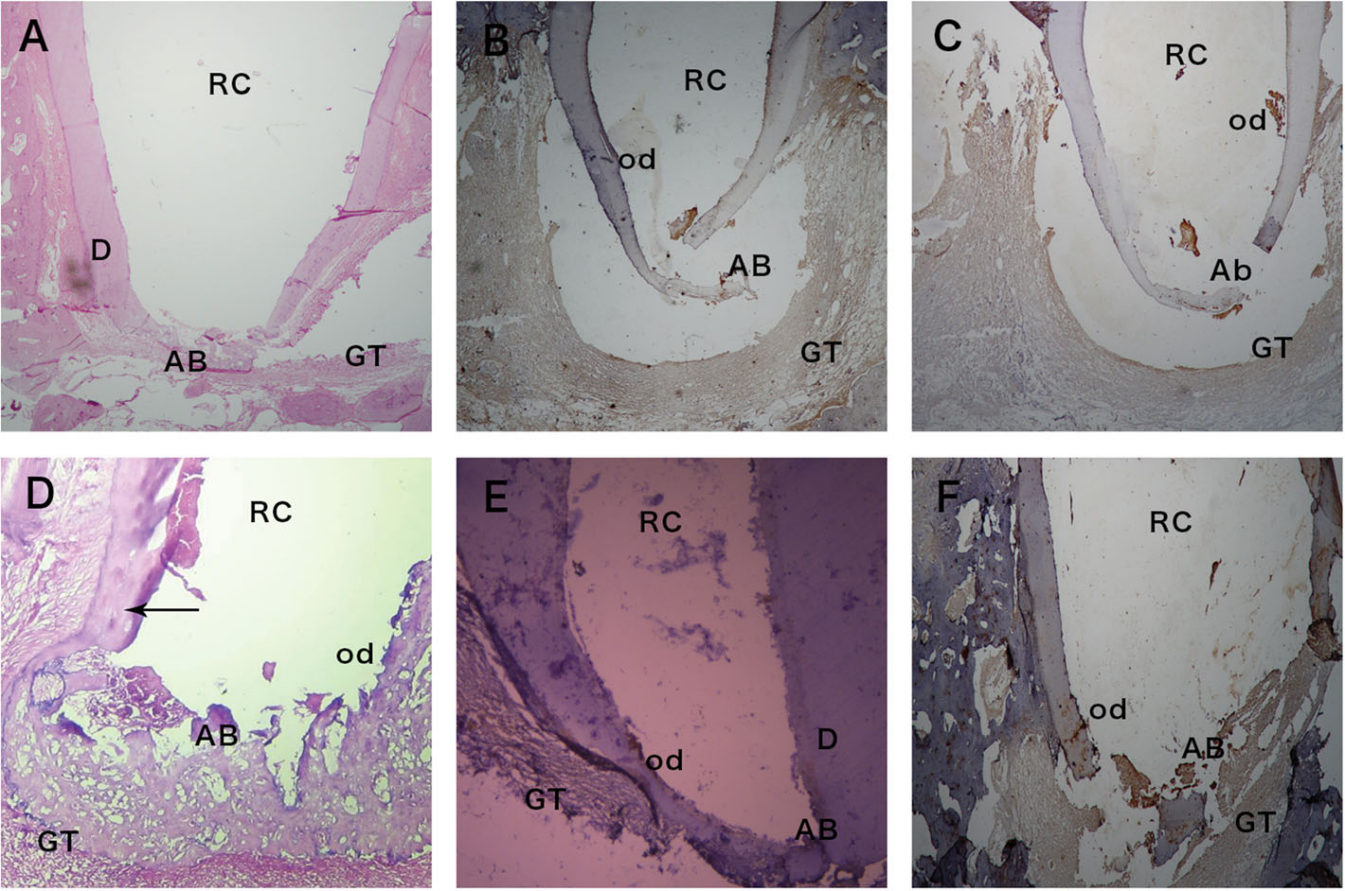
Premolar teeth treated with RAP or CH at 1-month. **a** Apical area of a canal treated with CH after 1-month showing the nearly complete closure of the root apices. Dentin (D), apical bridge (AB) granulation tissue (GT) surrounds the closed apex. Root canal (RC). H & E stain. Original magnification, × 100. **b** Apical area of a canal treated with CH after 1 month showing the nearly closed apex and apical bridge (AB) and odontoblasts (od) that show mild immuno-reactivity to the Wnt 10b antibody. Immuno-reactivity is also found in the granulation tissue (GT) apical to and at a distance from the regenerated dentin. Root canal (RC). Wnt10b antibody. Original magnification, × 100. **c** Apical area of a premolar tooth treated with CH after 1 month showing apical bridge formation (AB) showing mild immuno-reactivity to the nestin antibody. Immuno-reactivity is also observed in the odontoblast s (od) and the granulation tissue (GT) apical to and at a distance from the regenerated dentin. Root canal (RC). Original magnification, × 100. **d** Apical area of a premolar tooth treated with RAP after 1 month showing apical bridging (AB). Lateral walls thickened by newly formed dentin (arrow) show an odontoblast cell layer (od). Granulation tissue (GT) surrounds the closed apex. Root canal (RC). H & E stain. Original magnification, × 100. **e** Apical root region of a premolar tooth treated with RAP after 1 month showing complete apical bridging (AB) that is immuno-reactive to wnt10b antibody. Odontoblast cells (od) also show immuno-reactivity to WNt10b. Immuno-reactivity is also observed in the granulation tissue (GT) apical to the regenerated dentin. Dentin (D), root canal (RC). Wnt10b antibody. Original magnification, × 100. **f** Apical root area of a tooth treated with RAP after 1 month showing an opened apex that is bridged by cell condensations forming the apical bridge (AB) that shows immuno-reactive to the nestin antibody. Dense immuno-reactivity is also observed in the odontoblast cell layer (od) and granulation tissue (GT) apical to the cellular condensation and completely surrounding the opened apex. Root canal (RC). Nestin antibody. Original magnification, × 100

The outcomes of calcium hydroxide treatment were more variable over the post-treatment time intervals. At 1-month post-treatment, only few samples showed histological evidence of complete apical bridging (Fig. 1a, Tables 1 and 2), whereas most of the treated canals had root apices that were either wholly open or showed signs of incomplete closure (Fig. 1b & c). Mild staining for Wnt10b was identified in the newly formed dentin at the opened root apex and in the granulation tissue that was at a distance from root apex (Fig. 1b). Mild to moderate staining for the intermediate filament stem/progenitor cell marker nestin was identified in the newly formed dentin at the root apex. Granulation tissue showed moderate immuno-reactivity and was found at a distance from the root apex (Fig. 1c).

**Fig. 2:**
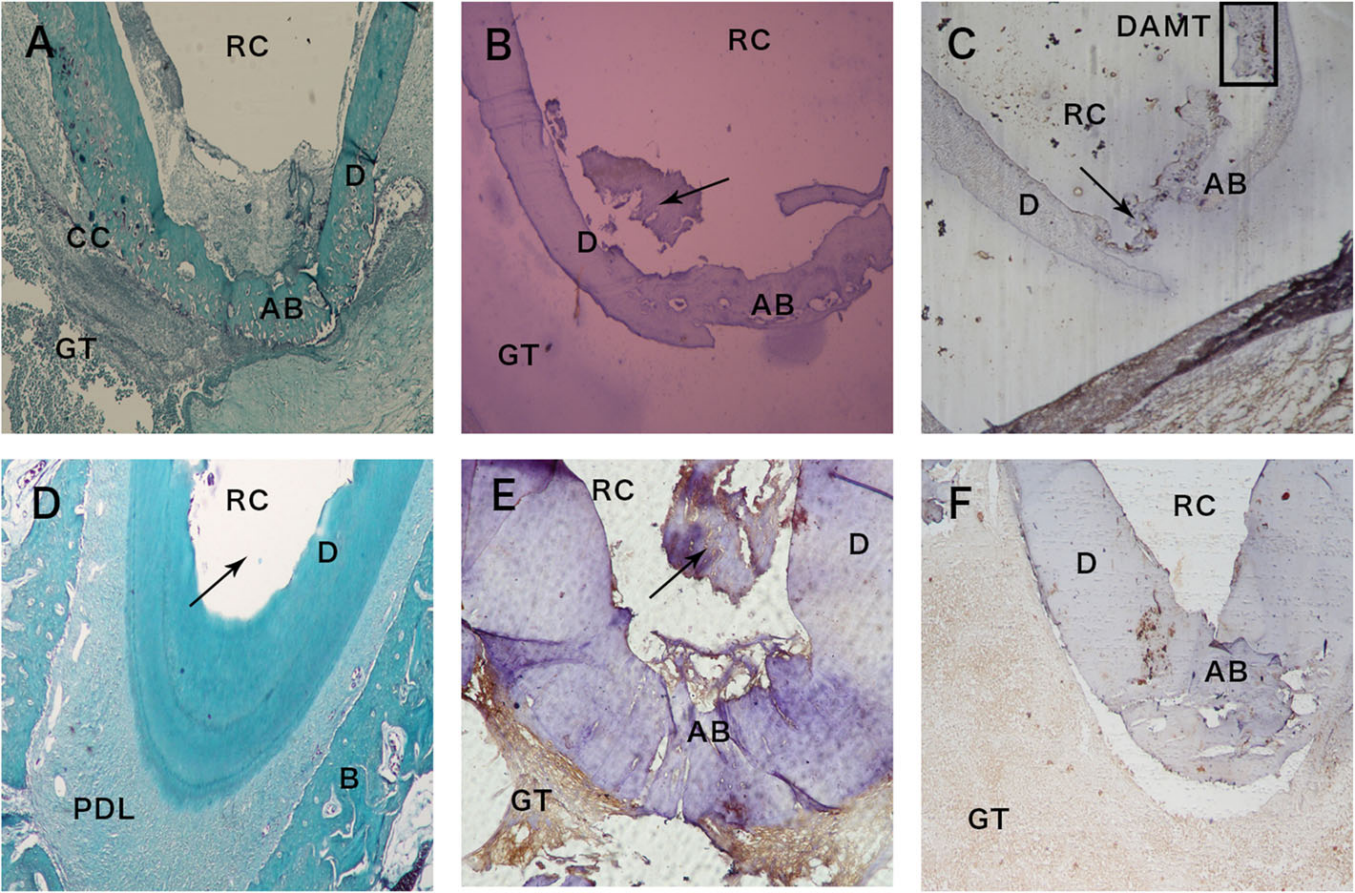
Premolar teeth treated with RAP or CH at 3 months. **a** Sagittal image of the apical region of the root of a premolar tooth treated with CH at the end of 3 months showing complete apical bridging (AB) of the root apices by cellular hard tissue formed of dentin (D) and cellular cementum (CC). Granulation tissue (GT) surrounds the closed apex. Root canal (RC). Trichrome stain. Original magnification, X 100. **b** Apical region of a premolar tooth treated with CH after 3 months of healing showing apical bridging (AB) of the open apex by a thin and almost complete layer of cellular hard tissue and dentin (D) that shows no immuno-reactivity to Wnt10b antibody. Granulation tissue (GT) also shows no immuno-reactivity to Wnt10b antibody. Some mineralized tissue (arrow) can be seen in the root canal (RC). Wnt10b antibody. Original magnification, × 200. **c** Apical root area of a premolar tooth treated with CH after 3 months showing incomplete apical bridging (AB) that shows immuno-reactivity to nestin antibody. Dentin-associated mineralized tissue (DAMT) (box). Odontoblast cells (arrow) and newly deposited dentin and DAMT show immuno-reactivity to the nestin antibody. Granulation tissue observed at a distance from the root apex (GT) also shows moderate nestin immuno-reactivity. Original magnification, × 100. **d** Apical area of a premolar tooth treated with RAP after 3 months showing apical closure achieved by tertiary dentin showing incremental lines (arrows). Periodontal ligament (PDL), bone (B), cementum (C) and root canal (RC). Trichrome stain. Original magnification, × 100. **e** Apical root area of a tooth treated with RAP after 3 months showing apical bridging (AB). Odontoblast cells (od). Some mineralized tissue (arrow) can be seen in the root canal (RC) and granulation tissue (GT) show intense immuno-reactivity to Wnt10b antibody Root canal (RC). Wnt10b antibody. Original magnification, × 200. **f** Apical root area of a premolar tooth treated with RAP after 3 months showing apical bridging (AB) that shows moderate to intense immuno-reactivity to the nestin antibody. Granulation tissue (GT) shows mild immuno-reactivity to nestin antibody. Root canal (RC). Nestin antibody Original magnification, × 100

At the 3-month interval, only few specimens of the canals showed complete root apex closure formed by cellular mineralized tissue (Fig. 2a, Tables 1 and 2). Most specimens showed incomplete root apex closure (Fig. 2c). No immune-reactivity was observed for Wnt10b either in the newly formed dentin or the granulation tissue (Fig. 2b). Mild staining for the intermediate filament stem/progenitor cell marker nestin was identified in the newly formed dentin and granulation tissue, while intense staining was observed in the odontoblast cell layer (Fig. 2c).

**Fig. 3:**
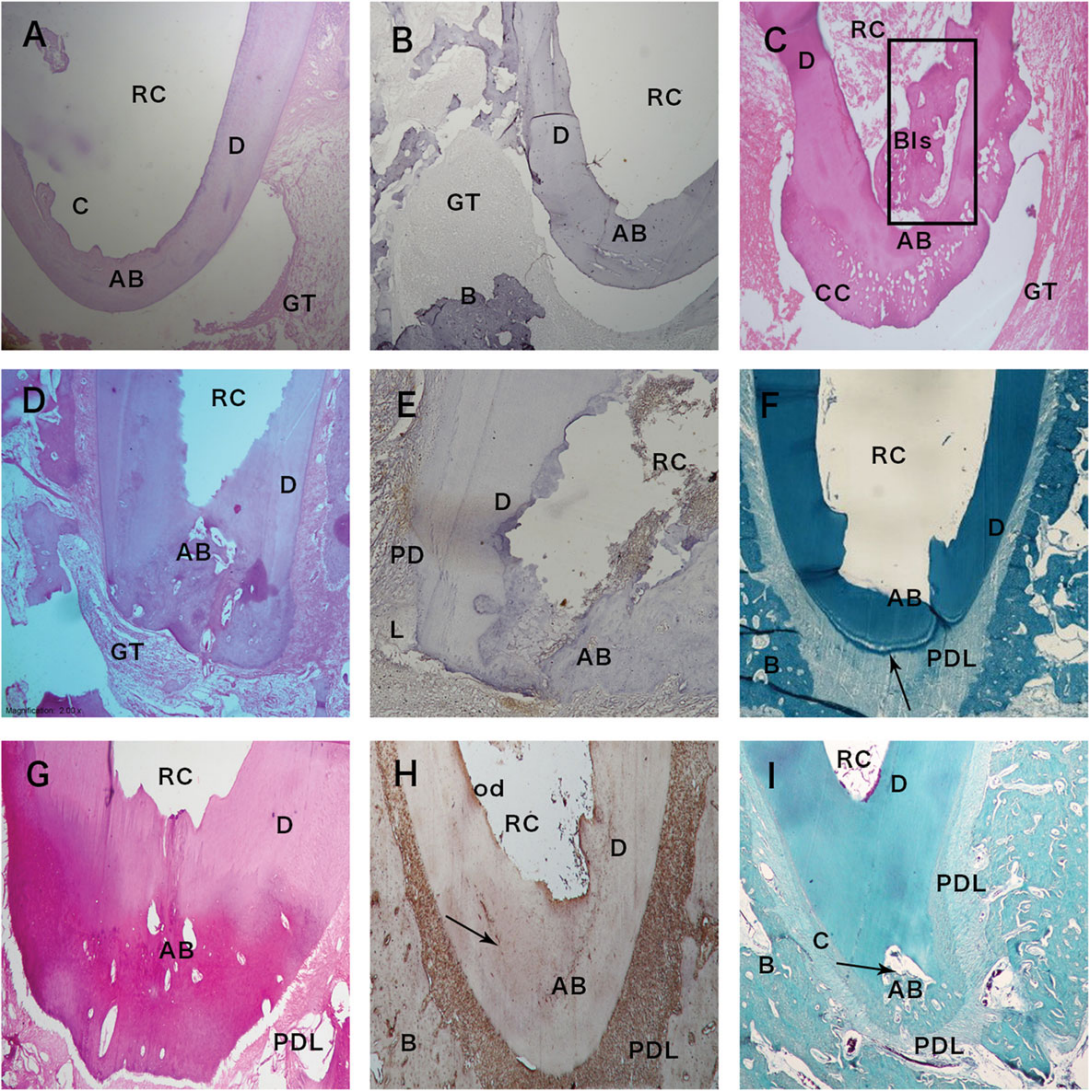
Premolars teeth treated with RAP or CH at 6 months. **a** Apical root area of a premolar tooth treated by CH after 6 months showing bridging of the apical foramen (AB). Granulation tissue (GT) surrounds and is observed at a distance from the regenerated root dentin. Root canal (RC). H & E stain. Original magnification, × 100. **b** Apical root area of a premolar tooth treated by CH after 6 months showing bridging of the apical foramen (AB). No immuno-reactivity to Wnt10bantibody is observed. Bone (B), granulation tissue (GT), root canal (RC). Original magnification, × 100. **c** Apical root area of a premolar tooth treated by CH after 6 months showing bridging of the apical foramen (AB) by calcified material and cellular cementum (CC). The root canal (RC) shows some calcified material in the apical root canal bony islands (BIs) (box), granulation tissue (GT). H&E stain. Original magnification, × 100. **d** Apical root area of a premolar tooth treated by RAP at 6 months showing bridging of root apex (AB) by cellular calcified material. Granulation tissue (GT) root canal (RC). H&E stain. Original magnification, × 100. **e** Apical root area of a premolar tooth treated by RAP at 6 months showing mild to moderate immunodetection of Wnt10b throughout the newly formed apical bridge (AB) and periodontal ligament (PDL). Root canal (RC). Wnt10b antibody. Original magnification, × 200. **f** Apical root area of a premolar tooth treated by RAP at 6-months showing apical bridging (AB) by a continuous layer of dentin (D) at the root apex. The newly formed dentin is attached to regenerated bone (B) by cementum (arrow) and periodontal ligament (PDL). Bone (B), root canal (RC). Trichrome stain. Original magnification, × 100. **g** Apical root area of a premolar tooth treated by RAP at 6 months showing bridging of root apex (AB) by cellular calcified material. Dentin (D), periodontal ligament (PDL) root canal (RC). H&E stain. Original magnification, × 200. **h** Apical root area of a premolar tooth root treated by RAP at 6-months showing bridging of the root apex (AB). Intense immunodetection of nestin antibody (Nesin) is observed in the newly formed apical bridge, odontoblast cell layer (od), in periodontal ligament (PDL) and in incremental lines in the tertiary dentin of the newly formed bridge (arrow). Regenerated bone (B) surrounds the regenerated root dentin. Root canal (RC). Wnt 10b antibody. Original magnification, × 100. **i** Apical root area of a premolar tooth root treated by RAP at 6 months showing the opened apex being bridged (AB) by cellular calcified tissue that extends into the root canal (arrow). The newly formed bridge is attached to regenerated bone (B) cementum (C) and periodontal ligament (PDL). Dentin (D) and Root canal (RC). Trichrome stain. Original magnification, × 100

At 6-months post-treatment, two histological patterns were recognized. The first was comprised of a thin layer with either a histologically incomplete or complete apical bridge (Fig. 3a & b, Tables 1 and 2). Minor thickening of the lateral portions of the root was also observed. Newly formed cementum was observed with no attachments to the granulation tissue. Incomplete islands of regenerated bone were observed, which had no attachments to the apical portion of the tooth. Granulation tissue persisted until the 6-months post-operative period. In the second pattern, canals showed complete apical closure by a continuous layer of mineralized tissue with thickened lateral walls. Mineralized tissue was present inside the apical portion of the root canal. In addition, the newly formed cellular cementum was not attached to the granulation tissue, which was always found at a distance from the apical bridge (Fig. 3c).

### Amelogenin-treated canal cohort

At one-month post-treatment, the canals treated with RAP showed the formation of dentin bridges, all of which were formed either by an incomplete or complete calcified tissue barrier (Fig. 1d & e, g, Tables 1 and 2). A consistent cellular response accompanying the apical closure was the appearance of a layer of cellular granulation tissue surrounding the root apex (Fig. 1d-f). The walls adjacent to the root apices appeared thickened due to the formation of newly formed dentin. Regenerated odontoblasts were found to be covering the newly formed dentin (Fig. 1d-e). Moderate to intense staining for Wnt10b was identified in the newly formed dentin at the root apex as well as among the odontoblast cell layer and in the granulation tissue (Fig. 1e). In addition, intense staining for nestin was recognized in the odontoblasts and in the condensed and regenerated cellular granulation tissue that completely surrounded the regenerating root apex (Fig. 1f).

At 3-months post-treatment, most of the amelogenin-treated teeth revealed complete root closure, as evidenced by a micro-anatomical analysis of the apical area, which showed cellular continuity and a dentin layer (Fig. 2d-f, Tables 1 and 2). Regeneration of the attachment apparatus was observed histologically in samples from this time interval (Fig. 2d). Wnt10b was recognized immunologically in the newly formed dentin and granulation tissue (Fig. 3e).

Mild to moderate to staining for nestin was recognized in the newly formed dentin bridge, and the granulation tissue (Fig. 2f).

At 6-months post-treatment with amelogenin, nearly all teeth showed histological evidence of the complete closure of the apical area by a thick layer of calcified tissue. A significantly higher percentage of amelogenin treated canals (70.9%) had a complete root closure in comparison to CH treated canals (10.8%) (*p*-value < 0.001) (Fig. 3d, Table 2). The presence of dentin-associated mineralized tissue was detected in a significantly higher percentage of roots treated with CH (43.3%) in comparison to none in roots treated with amelogenin (*p*-value< 0.001). The percentage of calcified islands were significantly higher in CH treated canals (23.3%) in comparison to amelogenin treated canals (5%) (*P* < 0.001). Dentin walls appeared to be thickened with newly formed dentine (Fig. 3d-i).

Moderate to intense staining for Wnt10b was recognized in the newly formed dentin and periodontal ligament (PDL) (Fig. 3e). Furthermore, intense staining to nestin was also recognized in these same structures (Fig. 3g).

In all of the teeth from the RAP group that did not show pulp regeneration, the newly formed dentin bridge was formed by tubular dentin (Figs. 2d-f, 3d-i).

## Discussion

An ideal endodontic treatment outcome for pulpal and periapical diseases is to reverse the destruction of local tissue and promote healing of apical periodontitis. In addition, in cases of immature teeth, promoting root development and restoring anatomical and physiological functions is warranted. Endodontic management of immature, non-vital, permanent teeth is a great challenge to dentists. The closure of the root apex is essential to achieve a complete, tridimensional sealing of the root canal system to prevent the entry of microorganisms or their products and increase the success of the endodontic treatment [16].

An immature tooth with an open apex is conventionally treated with apexification, requiring a long-term treatment with CH to induce the formation of an apical barrier. This long-term treatment weakens the root structure and exerts a cytotoxic effect on stem cells [4, 7]. Thus, one main cause of tooth loss following apexification is root fracture [17, 18]. Regenerative endodontics, is the extension of root canal therapy, it uses biologically based procedures to replace damaged tooth structures such as dentin, root structures and restore the apical seal. The optimal goal of regenerative endodontics is to regenerate the pulp tissue. The present study showed no pulp regeneration as revealed by absence of immuno-reactivity to nestin, which detects the neuronal derivatives of the neural crest that comprises the dental pulp [19, 20]. Treatment modalities will change in teeth with regenerated roots and regenerated pulps as compared to teeth with regenerated roots and no regenerated pulps. The RAP treated canals showed a calcified tissue barrier forming a natural obturation that sealed the empty root canal cavities from the periapical area. The lateral dentin wall and apex thickening lined by the newly formed odontoblasts resultes in strengthening of the canal’s lateral wall, which is one of the main disadvantages of apexification [21, 22], further studies must be conducted to investigate whether these teeth must be filled with a suitable canal filling material or left without filling for further lateral dentin deposition. Cellular destruction in the wound area that occurs as a result of infection of the root canal and periapical area, observed as bone destruction around the root apices, could be healed via amelogenin therapy [14].

No intra canal medicament was used when teeth were cleaned, irrigated, dried and closed for one week to emphasize the presence of infection, and how the recombinant protein can regenerate the lost tissue in the presence of infection. The granulation tissue observed in the early phases of wound healing surrounding the apical region of the healing roots in both treated groups showed nestin immuno-reactivity; however, this intensity was increased in the amelogenin group, especially at one month. The stem/progenitor cells of the apical papilla and all odontogenic ectomesenchymal cells have been shown to express the nestin neurogenic marker [23]. This may indicate that this tissue can develop into apical-papilla stem cells (APSCs) [24, 25]. Wnt 10b-mediated activation of canonical Wnt signaling has been shown to regulate mesenchymal stem cell fate by promoting regeneration via local MSC/progenitor cell recruitment. LRAP induces cementum and bone formation in human stem cells through the activation of the Wnt signaling cascade [26]. In our study, the apical root areas treated with RAP for 6 months showed moderate to intense immuno-reactivity to Wnt 10b in the newly formed cementum, periodontal ligament, and bone that constituted the newly formed attachment apparatus. This may indicate that the regenerative tissue products are, at least partly, mediated by the activation of canonical Wnt signaling pathway.

Yamuchi et al. reported two types of mineralized tissues being formed in the root canal space in experimental animals after undergoing tissue-engineering therapy: dentin-associated mineralized tissues (DAMTs) and bony islands (BIs) [27]. In the present study, DAMTs and BIs were mainly observed in the CH group. The presence of DAMTs and BIs may complicate root canal filling, thus implicating the favor of using amelogenin as material of choice. At 6 months, the apices all canals that showed no pulp regeneration were completely closed. Although the root canal was empty, it still showed regular outlines and had relatively thick dentin, which was attached to newly formed bone by newly regenerated periodontal ligament fibers. This created a natural obturation that sealed the root canal cavity from the periapical area. As mentioned before, further studies must be conducted to investigate whether these teeth must be filled with a suitable canal filling material or left without filling for further lateral dentin deposition. If further studies were in favor of root canal filling, the histological results in this study support the use of root canal filling with a material capable of adapting to the canal anatomy, especially in the apical third of the canal.

## Conclusions

Recombinant amelogenin shows great potential as an apexification agent alternative to traditional CH therapy. It provides an alternative technique for root canal filling, in which the regenerated calcified barrier can form a natural obturation, stimulating root growth and the regeneration of the dental attachment apparatus in non-vital, young, permanent teeth.

## Abbreviations

AB: Apical bridge
B: Bone
BIs: Bony islands
C: Cementum
CC: Cellular cementum
CH: Calcium hydroxide
CIs: Calcified islands
D: Dentin
DAMT: Dentin-associated mineralized tissue
GT: Granulation tissue
Od: Odontoblasts
PDL: Periodontal ligament
RAP: Recombinant amelogenin protein
RC: Root canal
